# The impact of childhood diagnosed ADHD versus controls without ADHD diagnoses on later labour market attachment—a systematic review of longitudinal studies

**DOI:** 10.1186/s13034-021-00386-2

**Published:** 2021-06-23

**Authors:** Marie Sturesson Christiansen, Merete Labriola, Lilli Kirkeskov, Thomas Lund

**Affiliations:** 1 Center for Social Medicine, Bispebjerg and Frederiksberg Hospital, Copenhagen, Denmark; 2 NORCE, Norwegian Research Centre AS, Bergen, Norway; 3 Department of Public Health, University of Copenhagen, Copenhagen, Denmark

**Keywords:** Attention Deficit/Hyperactivity Disorder, Life-course, Occupation, Work, Employment

## Abstract

Previous studies have suggested that Attention Deficit Hyperactive Disorder (ADHD) affect individuals across their lifespan, especially in relation to employment. The purpose of this review was to synthesize the results from studies examining the prospective association of ADHD diagnosis in childhood and later education, earnings and employment, compared to children without an ADHD diagnosis. A review protocol was prospectively registered with PROSPERO (ID = CRD42019131634). The findings were reported according to the PRISMA guidelines. The systematic review is based on a structured and preplanned analysis of original prospective longitudinal studies. A total of 2505 potential records were identified, two through backward search. Six papers met the inclusion criteria. One paper was assessed as good, four as fair and one as poor quality. The studies indicated that ADHD diagnosis affected the nature of the individual’s attachment to the labour market across different labour market attachment outcomes. Adults with persisting symptoms, had significantly more problems at work. Even if ADHD symptoms desist in adulthood, the negative impact of earlier ADHD symptoms can still be seen on occupational outcomes. Significantly fewer probands had a Bachelor’s degree compared to controls. Based on one good quality study and four fair quality studies, it is indicated that patients with childhood diagnosed ADHD, generally experience employment of lower quality compared with peers, in relation to income, education and occupational attainment. The overall level of evidence is rated as poor.

**Supplementary Information:**

The online version contains supplementary material available at 10.1186/s13034-021-00386-2.

## Background

Attention Deficit Hyperactive Disorder (ADHD) is the most frequently registered psychiatric diagnosis among children and adolescents, and the diagnosis is three times more prevalent among boys than among girls [4]. Girls are more often diagnosed with Attention Deficit Disorder (ADD), due to absence of hyperactivity [17]. It is estimated that the worldwide prevalence of ADHD/ADD is 5.3% [14]. Based on a review from 2014 there has been no change in the prevalence across the three last decades [15]. In Denmark, in contrast to the review by Polanczyk et al. [15], the prevalence of ADHD among children and adolescents (0–18 years) has more than tripled from 2006 to 2016, resulting in 25,029 children and adolescents diagnosed with ADHD in 2016. The incidence was almost doubled in the same period, with 4128 children and adolescents diagnosed with ADHD in 2016 [5]. The reason for the increased prevalence of ADHD, in Denmark, may be due to a greater awareness of ADHD and more children psychiatrist to handle the diagnostics.

ADHD is diagnosed according to the DSM-5 (Diagnostic and Statistical Manual of Mental Disorders by American Psychiatric Association) [1] and the ICD-10 (International Classification of Diseases) of the World Health Organization [19] when inattentiveness, hyperactivity and impulsiveness are present in childhood.

Although most frequently diagnosed during the school years, ADHD is now acknowledged to affect individuals across the lifespan [2]. ADHD is a chronic disorder, that may have important impact on school achievement, education and on the individual’s life course [7, 18]. Around 65% of children with ADHD still have problems in their adult life [18]. Symptoms have been observed to change over time, and the hyperactivity component may decline during adolescence. Inattention symptoms tend to persist into adulthood [6]. Children with ADHD may have concentration- and attention problems, which can result in difficulties in following a normal school program and result in familial problems (e.g., parents, siblings etc.) [7]. Children with ADHD have an increased risk of dropping out of school, and adolescents with ADHD may have difficulties in completing an academic education [18]. Based on this, youths with ADHD seem to achieve a lower level of education and that this may have an impact on the future labour market attachment.

Our hypothesis is that ADHD can affect learning and psychological wellbeing and negatively affect education and later labour market participation. Given the high prevalence of ADHD, this would imply that a large group of youths will not reach the same economic and educational level as peers.

The aim of this study is to describe existing knowledge on prospective associations between ADHD in youth (6–17 years old) and future employment.

## Methods

A review protocol was prospectively registered with PROSPERO (ID = CRD42019131634). The findings were reported according to the PRISMA guidelines for systematic reviews.

A systematic review was performed of prospective longitudinal studies addressing the impact of childhood ADHD diagnosis on later labour market attainment.

### Search strategy

MEDLINE, Web of Science, PsycINFO and The Cochrane Library (Cochrane Database of Systematic Reviews) were used for systematic searching in December 2018 and again in November 2020. A research librarian helped develop the search strategy. The search terms used were “Attention Deficit Disorder with Hyperactivity”, “attention deficit disorder” or “ADHD”, combined with “adult” and “occupations”, “work”, “employment” or “workplace”.

### Study selection

References were imported into Covidence. Title and abstract screening, full-text screening and data extraction were performed independently by two of the authors of this paper. The inclusion criteria were set to include quantitative prospective longitudinal studies, in English, Danish, Swedish and Norwegian. The study population included adults who were diagnosed with ADHD/ADD at age 6–18 years and comparable ADHD/ADD-free controls. No timeframe was set. Studies performed in low- and middle-income countries were excluded in order to increase homogeneity of labour market regimes. If the population included adults not in working age (> 60 years), the study was also excluded. All the included studies needed to have age- and sex-matched comparison participants without ADHD/ADD. Finally, backward searching of reference lists of the located studies were performed. References in systematic reviews on similar topics were also screened to localize further relevant studies.

### Quality appraisal

Quality Assessment Tool for Observational Cohort and Cross-Sectional Studies, from the National Heart, Lung, and Blood Institute [12] was used for assessing the quality of the studies and risk of bias. Quality assessment was independently conducted by all the authors. The first author (MSC) assessed all selected articles and the three other authors assessed a third each. Each article was classified as “good”, “fair” or “poor”. Any disagreements were resolved in collaboration and by discussion with a 3rd person.

### Data extraction

The first author (MSC) used a data extraction sheet, to extract data from each of the included studies. Afterwards the second author (ML) checked the data. Disagreements were solved through discussion. The collected information included author name, publication date, objectives, study design, study period, follow-up, population, inclusion/exclusion criteria, variables of interest, measurement, statistical methods, participants, confounders, outcome data, main results and limitations.

## Results

A PRISMA flow diagram of the search results and reasons for study exclusion can be found in Additional file 1: Figure S1.

The initial literature search provided 2505 references. After removal of duplicates, 2028 references were left for title and abstract screening. Of these, 30 references were relevant for full text screening, and of these four articles were included. Backward searching of reference lists of the located studies, provided two more relevant articles. Screening of the studies included in previous reviews on similar topics did not provide further relevant articles. The search was repeated in November 2020, yielding an additional 15 studies eligible for screening. No additional studies were included following abstract and full-text screening. The search yielded a new systematic review with relevance for this review, but the studies included did not fulfill the inclusion criteria for the present review.

Table 1 summarizes the characteristics of the six studies meeting inclusion criteria. All studies used DSM as diagnostic criteria to identify the ADHD study population. All studies included measures related to occupation and education at follow-up [3, 8, 10, 11, 13, 16], five studies included information on financial follow-up outcomes [3, 8, 10, 13, 16]. Two studies included follow-up information of general socioeconomic position [3, 10].

**Table 1:** Characteristics of the six studies

Study, country	Number of subjects	Age at first assessment	Mean age at follow up	Participants (eligible, included, completed follow-up) (%)	Study age range	Collection of outcomes at last follow-up	Outcome measures	Results
Roy et al. [16], USA, Canada, Germany	579 children with ADHD and 258 controls	7–10 years	24.7 years	476 (82%) with ADHD and 241 (93%) controls	16 years after baseline	Questionnaire performed by participants and parents	Occupation:Job changes (total number of times fired or quit) Earnings: Public assistance (yes/no) Income levels (measured on a scale of 1–10) Education:Bachelor’s degree (yes/no)	ADHD severity predicted an increased likelihood of more job changes (number of times fired or quit) and receiving public assistance ADHD severity predicted reduced likelihood of receiving a bachelor´s degree
Owens et al. [13], USA	140 girls with ADHD and 88 controls	6–12 years	25.6 years	123 (88%) with ADHD and 85 (97%) controls	16 years after baseline	Clinic-based assessment, structured interview. Parents completed questionnaires	Occupation: Life-RIFT, Problems at work, Current employment status, hours worked per week Earnings: Receiving public assistance, current monthly salary Education: Highest degree, years of education	No statistically significant differences among any groups on current employment, hours worked per week, receiving public assistance or current salary Persisters (participants who met ADHD criteria in all study waves) showed significantly more problems at work. Comparisons reported significantly better functioning at work than girls with persistent ADHD. Overall, employment outcomes among comparisons were slightly better than desisters and partials (participants who met the criteria for ADHD at baseline (W1) and at third (W3) or forth (W4) study wave), but much better than persisters Probands had finished 2 less years schooling
Hechtman et al. [8], Canada, USA	579 individuals with ADHD and 258 controls	7–10 years	24.7 years	476 (82%) with ADHD and 241 (93%) controls	16 years after baseline	Questionnaire performed by participants and parents	Occupation: Number of jobs, average job length, number of times fired or quit Earnings: Current income, receiving public assistance (yes/no) Education: Bachelor´s degree (yes/no)	Significant difference in average job length, number of times fired or quit, current income and public assistance 16.0% of ADHD participants vs. 3.2% of controls received public assistance. Persisters showed the worst outcomes and the controls the best. 22.2% of persisters and 9.6% of desisters received public assistance Majority of ADHD group (61.7%) had a high school degree or less. Majority of control (60.8%) group had completed at least some college. 8.0% of persisters had a Bachelor´s degree, 17.8% of desisters and 37.1% of controls
Klein et al. [10], USA	207 boys with ADHD and 178 controls	6–12 years	41.4 years	135 (65%) with ADHD and 136 (76%) controls	33 years after baseline	Interviews performed of trained and closely supervised doctoral level clinical psychology candidates	Occupation: Hollingshead (scaled 1–8) (jobs held, job satisfaction, work relationships, latenesses, job changes, firings), Occupational function scaled 1–6) Earnings: Annual salary SES: 5-point scale (high score = low SES) Education: Years of education, highest degree	ADHD group had significantly lower occupational attainment levels, but 84% of them were holding jobs. Employed probands had an annual salary $40,000 beyond the controls Employed probands had "average" to "good work" performance. Comparisons were significantly superior (good-very good) Probands had 2.5 fewer years of schooling then controls. Significant fewer probands had a Bachelor´s degree (16%) vs. controls (35%)
Biederman et al. [3], USA	140 with ADHD and 120 controls	6–17 years	27.1 years	79 (56%) with ADHD and 90 (75%) controls	16 years after baseline	Structured clinical interviews performed of interviewers that had undergraduate degrees in psychology and were extensively trained Interviewed all subjects and their mothers	Occupation: Hollingshead, Occupational rankings 1–9 Earnings: Financially dependent on their parents SES: Hollingshead 5-point scale. High score = Low SES Education: Educational level Hollingshead scale 1–7 , College graduate yes/no	Significantly lower occupational level in the ADHD group compared to controls Men how had ADHD as boys were significantly more likely to be financially dependent on their parents and had lower social class. Boys with ADHD had significantly lower personal socioeconomic status vs. their family of origin socioeconomic status They were also less likely to graduate from college
Mannuzza et al. [11], USA	103 boys with ADHD and 100 controls	6–12 years	25.5 years	91 (88%) with ADHD and 95 (95%) controls	13–19 years after baseline	Semi-structured psychiatric interviews performed of a doctor-level clinical psychologist and a psychiatric social worker	Occupation: Hollingshead Occupational rankings 1–7. Owners of small business Education: Years of formal schooling completed, Highest degree completed, Bachelor’s degree (yes/no)	90% of both probands and controls were employed at follow-up, but probands significantly lower occupational rankings. 18% of probands and 5% of controls were owners of small businesses Probands had significantly 2.5 fewer years of schooling then controls. Significant fewer probands had a Bachelor´s degree or higher (12%) vs. controls (49%)

The six studies were published between 1993 and 2017 (median 2015). The combined study populations of the included studies totaled 2268 individuals, of which 1380 were probands and 888 were controls. However, two studies [8, 16] were based on the same study population. Thus, the true size of the total population under study was 904 probands and 647 controls, totaling 1551 individuals. Overall, this indicates generally small to medium sized studies, ranging from 169 individuals [3] to 717 individuals [8, 16]. All the studies were based on identification of probands in clinical or educational settings and age- and gender matched ADHD-free controls [3, 8, 10, 11, 13, 16]. Follow-up periods ranged from 16 [8, 16] to 33 years [10]. None of the included studies addressed ADD directly, but one study included one person with ADD [11].

### General description of study participants

Age at inclusion of study participants ranged from six to seventeen years of age. Two studies did not report gender distribution but included both females and males, n = 717 for both studies [8, 16]. Of the remaining four studies, one study exclusively included females (n = 208) [13] and three studies exclusively included males, (Klein et al. n = 271, Biederman et al. n = 169, Mannuzza et al. n = 186) [3, 10, 11].

### Assessment of quality of included studies

The quality appraisal of the 6 papers resulted in one paper assessed as good quality [13], four as fair quality [3, 8, 11, 16], and one as poor quality [10] (Table 2). The four authors were in agreement in 50% of the classifications into good, fair and poor quality categories. Based on the quality assessment, only the results from the five studies rated fair or good quality [3, 8, 11, 13, 16] were included in the review results (Table 3).

**Table 2:** Quality assessment

Study	Good	Fair	Poor
Roy et al. [16]		✕	
Owens et al. [13]	✕		
Hechtman et al. [8]		✕	
Klein et al. [10]			✕
Biederman et al. [3]		✕	
Mannuzza et al. [11]		✕

**Table 3:** Quality ratings of included studies

Study	Clearly stated research question/objective	Clearly specified/defined population	Participation rate ≥ 50%	Population, inclusion/exclusion criteria	Sample size justification	Exposure measured prior to the outcome	Timeframe	Levels of exposure	Valid and reliable measurement of exposure	Assessment of exposure more than once	Valid and reliable measurement of outcome	Blinded outcome assessors	Loss to follow-up ≤ 20%	Measured and statistically adjustment of confounding	Rating
Roy et al. [16]	Yes	No	NR	No	No	Yes	Yes	No	Yes	No	Yes	No	NR	Yes	Fair
Owens et al. [13]	Yes	Yes	Yes	Yes	Yes	Yes	Yes	Yes	Yes	Yes	Yes	Yes	Yes	Yes	Good
Hechtman et al. [8]	Yes	No	NR	No	No	Yes	Yes	Yes	Yes	Yes	Yes	No	Yes	Yes	Fair
Biederman et al [3]	Yes	Yes	CD	Yes	No	Yes	Yes	NR	Yes	Yes	Yes	Yes	No	Yes	Fair
Mannuzza et al. [11]	CD	Yes	Yes	No	No	Yes	Yes	No	Yes	No	Yes	Yes	Yes	Yes	Fair

Due to low quantity of included studies, heterogeneous outcomes and generally low quality of the included studies, meta-analysis and test for heterogeneity was not deemed feasible.

### Labour market attachment

Occupational attainment was assessed using different measures; (un)employment, number of jobs, average job length, times fired or quit, employment level/rank, occupation/industry.

#### Job changes

Two studies used self-reported job changes “total number of times fired or quit” [8, 16]. Roy et al. found that number of times fired or quit was predicted by a high baseline ADHD severity score (OR = 1.20, p < 0.001) [16]. Hechtman and colleagues found no significant differences between number of jobs held among probands (2.2, SD = 1.3) and controls (2.1, SD = 1.3) (p = 0.08). ADHD probands had experienced twice as many job changes due to having quit or being fired (0.61 SD = 1.06 vs. 0.32 SD = 0.64, p < 0.001), and had a significantly shorter average job length (381 SD = 341 vs. 422 SD = 325, p < 0.001). No unit for the measure of job length was provided [8].

#### Problems at work

Information about problems at work was obtained using a project-derived structured interview. Owens et al. found that girls with persistent ADHD across all study waves showed significantly more problems at work than the other groups (desisters and controls, ds = 0.69–0.94) [13]. According to self-report, employment functioning was essentially equivalent across groups, with the exception that controls reported significantly better functioning at work than girls with persistent ADHD (d = 0.68). Overall, employment outcomes among comparisons were slightly better than desisters [13].

#### Occupational rankings

Two studies used occupational rankings according to Hollingshead et al. [3, 9, 11]. Mannuzza and colleagues found that about 90% of subjects in each group were employed at follow-up, but probands had significantly lower occupational rankings than those of controls (ranking 3.5 vs. 4.4; P < 0.0001) [11]. Likewise, Biederman and colleagues found occupational level significantly lower within the ADHD group compared to controls (mean 5.2 vs. 6.6 *p* = 002, scale score range 1–9), also after controlling for total number of psychiatric disorders other than ADHD [3]. In order to study potential differences in the type of work adults with or without ADHD were employed in, Mannuzza and colleagues also investigated differences in job type between probands and controls, and found the largest discrepancy occurred among the higher-ranking positions, e.g., 21% of controls vs. only 4% of probands were employed as professionals (P < 0.001). Conversely, group rates were comparable for lower-ranking positions and among skilled workers. Whether they owned and operated their own business was also reported, and it was found that 18% of the probands and 5% of controls were owners of small businesses [11].

The ADHD diagnosis seems to affect the nature of the individual’s attachment to the labour market [3, 8, 11, 13, 16] across different labour market attachment outcomes.

### Earnings

#### Public assistance and income

Four studies have investigated the economic situation in adulthood of participants with a history of ADHD in childhood [3, 8, 13, 16]. In the study by Hechtman and colleagues it was found that 16% of probands received public assistance, where it was 3.2% of controls [8]. The comparisons of the ADHD subgroups relation to previous year income showed that the subgroup with persistent symptoms showed the worst outcomes, the symptom-desistent subgroup intermediate and controls the best [8].

Roy and colleagues found significantly increased likelihood (OR = 1.50 SE = 0.13 *p* = 0.02) of receiving public assistance in adulthood, with higher baseline ADHD symptom severity, compared to ADHD free controls [16].

Among 140 girls with ADHD and 88 age- and ethnicity-matched comparison girls*,* Owens and colleagues used ANOVAs and eight chi-squared tests and found no statistically significant difference between probands and controls in relation to receiving public assistance [probands 14.1% controls 38.0% (*p* = 0.012)], or in current salary levels [probands 2087 (SD = 1896) controls 1167 (SD = 1420) (*p* = 0.023)] [13]. Finally, men who had ADHD as boys were significantly more likely to be financially dependent on their parents compared to controls (26.6% vs. 13.3% *p* = 0.03) [3]

#### Socio-economic position

Biederman et al. was the only study that reported Socioeconomic Status (SES) at follow-up (16 years follow-up). SES was measured using the 5-point Hollingshead scale, with high score equaling low SES. At follow-up, the mean age was 27.4 years old. Biederman and colleagues followed 79 boys with ADHD and 90 controls and found that participants with ADHD in childhood that were financially independent, had significantly lower personal SES (mean 1.7 SD = 0.8) than controls (mean 1.4 SD = 0.6) (*p* = 0.02) at follow-up [3].

### Educational attainment

All the five included studies comprised measures of educational attainment at follow-up. These can be roughly divided into two main categories: educational achievement [3, 8, 11, 13, 16] and obtained college/university degree [3, 8, 11, 16]. In addition to this, one study addressed educational functioning [13].

#### Educational achievement

Owens and colleagues found higher educational degrees among controls than among ADHD cases: On a 6-point scale, the group most affected by ADHD had a score of 1.7 (SD 1.1) versus 3.2 (SD 1.4) among the controls (p < 0.001) [13]. This finding was confirmed by the findings by Hechtman and colleagues, who showed that 61.7% of probands had high school or less, where this was the case for 39.2% of controls [8]. Proportions with college or trade education as highest degree was 23.2 percent among probands and 18.8 among controls. Among controls 42.1% had a bachelors or master’s degree—this was the case for 15.1% of the probands. The unadjusted association was found to be statistically significant (p < 0.001) [8]. Biederman and colleagues used the Hollingshead scale to analyze potential differences in educational level. On a scale score range from 1 to 7 where higher values indicated higher educational levels, controls had a mean score of 6.1, probands 5.1 (p < 0.001) [3]. Mannuzza and colleagues showed analyses compatible to the study by Hecthman and colleagues [8, 11]: The proportion with high school or less was 63% among probands and 27% among controls, and probands had finished 2 years less schooling than controls (p < 0.001) [11] (Table 4).

**Table 4:** Educational achievement

		ADHD	Controls
Owens et al. [13]	Highest degree earned	1.7^a^	3.2^a^
Hechtman et al. [8]	Bachelor degree or master degree	15.1%	42.1%
Biederman et al. [3]	College graduate	37.9%	84.6%
Mannuzza et al. [11]	Bachelor degree	9%	34%

^a^6-point scale

﻿

#### Obtained college/university degree

Three papers addressing associations between ADHD and receiving a university degree were based on two underlying studies: Two papers were based on The Multimodal Treatment Study of Children with ADHD [8, 16] and one was based on children referred to the same no-cost child psychiatry clinic [11].

According to Roy and colleagues, the chance for probands to obtain a bachelor’s degree decreased with ADHD symptoms severity measured with the SNAP (Swanson, Nolan and Pelham) scale, where a 1-step increase on the 4-category SNAP scale assessed at baseline had an OR of 0.69 (SE = 0.11; P = 0.02) for obtaining a bachelor’s degree in adulthood [16]. Based on the same data material, Hechtman and colleagues confirmed this association: ADHD-free controls had a OR of 1.29 for obtaining a bachelor’s degree during follow-up compared to probands [8]. Biederman and colleagues found a comparable educational gradient, as 84.6% of controls had obtained a bachelor degree at follow-up, where this was the case for 37.9% of probands (p < 0.001) [3].

Mannuzza and colleagues found similar associations among children referred to a child psychiatric clinic and diagnosed with ADHD, compared to a ADHD-free group of matched controls: 12% of probands had complete a bachelor’s degree or higher at follow-up, compared to 49% of controls (p < 0.001) [11].

#### Educational functioning

Owens and colleagues showed higher educational functioning scores among controls than among probands. The educational functioning was reported by the participants, clinicians and the participants parents. All groups had the highest educational functioning score among controls, and the score was lowest among the “persisters” (participants who met ADHD criteria at all waves) [13].

## Conclusions

Of the six studies meeting inclusion criteria, one was rated of good quality, four of fair, and one of poor quality. Based on the five studies rated fair or good, it is indicated that patients with ADHD generally experience employment of lower quality (lower lifetime income, more prone to part time and unskilled work) and are more likely to receive public assistance.

While ADHD is shown to be a significant negative predictor for future occupational outcomes, other factors such as socioeconomic status and test scores can work opposite to this. This suggests that adult outcomes for ADHD patients can be affected. However, it is currently not evident from existing literature, included in this article, which factors positively affect educational and occupational attainment. Given the significant size of the patient population, finding ways to better adult outcomes would have large implications. Overall, the scarcity and quality of the studies meeting inclusion criteria suggest that the level of evidence for the associations targeted in this systematic review is deemed as poor. This should warrant further research addressing which factors and interventions help reduce the long-term vocational effects of childhood ADHD and especially ADD which is not addressed in the present literature.

## Supplementary Information

**Additional file 1: Figure S1.** Search results and reason for study exclusion.

## Data Availability

Not applicable.

## Abbreviations

ADHD: Attention Deficit Hyperactive Disorder
ADD: Attention Deficit Disorder
DSM-IV: Diagnostic and Statistical Manual of Mental Disorders by American Psychiatric Association
IDC-10: International classification of diseases
SES: Socioeconomic status
SNAP scale: Swanson, Nolan and Pelham scale
