# Supplementary material for: The impact of childhood diagnosed ADHD versus controls without ADHD diagnoses on later labour market attachment—a systematic review of longitudinal studies
Source: Child Adolesc Psychiatry Ment Health. 2021 Jun 23;15:34. doi: 10.1186/s13034-021-00386-2 (PMC8220843; doi:10.1186/s13034-021-00386-2)
Supplement: Supplementary file 1 — Additional file 1: Figure S1. Search results and reason for study exclusion. [file 13034_2021_386_MOESM1_ESM.docx]

Additional file 1

Figure S1

References identified through online databases in December 2018
(n = 2505)

References screened against title and abstract
(n = 2028)

Duplicates removed
(n = 477)

Full-text articles excluded

(n = 26)

Reasons: 7 adult population

7 wrong study design

9 wrong patient population

2 book

1 wrong comparator

References excluded
(n = 1998)

References assessed for full-text eligibility

(n = 30)

References included

(n = 4)

Total references included in review

(n = 6)

Backward searching of reference lists

(n = 2)

New references identified through online databases in November 2020
(n = 90)

References screened against title and abstract
(n = 15)

References included

(n = 0)
